# Outcomes in Patients with Untreated Versus Treated Asymptomatic Bacteriuria within 5 Veterans Affairs Facilities

**DOI:** 10.1017/ash.2024.154

**Published:** 2024-09-16

**Authors:** Kelly Davis, Jessica Bennett, Angela Kaucher, Bowden Jarred, Brittany DeJarnett, Garrett Fannin, Morgan Johnson, Anna Mitchell, Colby Osborne, Caroline Powers, Caroline Williams, Dana Williams, Hans Scheerenberger

**Affiliations:** Lexington VA HealthCare System; Lt. Col Weathers Jr VA Medical Center; James H. Quillen VA Medical Center; Lt. Col. Luke Weathers, Jr. VA Medical Center; Robley Rex VA Medical Center; Tennessee Valley Healthcare System; Department of Veterans Affairs; Ralph H. Johnson VA Medical Center

## Abstract

**Background:** Asymptomatic bacteriuria (ASB) is often treated with antibiotics despite recommendations against screening for and treating ASB in most populations. Some providers cite concern for progression of ASB to a symptomatic urinary tract infection (UTI) as the jultification for antibiotic use. While the 2019 Infectious Diseases Society of America (IDSA) ASB guidelines refute this concern, most evidence is derived from studies done in females, potentially limiting external validity. The purpose of this study is to compare the outcomes of patients with ASB who received antibiotic treatment versus those who did not in a primarily male population. **Methods:** This is a multi-center, retrospective, cohort study conducted by the 5 sites within the Veterans Affairs MidSouth Healthcare Network. Patients with a positive urine culture (defined as cultures with a colony forming unit count >100,000) collected from January 1, 2021 through December 31, 2021 were identified. ASB was determined via chart review using pre-determined criteria (positive culture in the absence of reported or documented signs or symptoms attributable to UTI as defined by the 2019 IDSA ASB guidelines). Additional data collected included antibiotic use, clinic visits and hospital admissions related to UTI or sepsis from a UTI. The primary outcome was the comparison of UTI incidence at 30 days, 6 months, and 1 year in those untreated versus treated with antibiotics. Secondary outcomes included a comparison of admissions with sepsis from UTI and adverse drug reactions (ADRs) between the cohorts. Continuous data were analyzed using a Student’s t-test. Discrete data were analyzed using either a Chi-squared or Fisher’s exact test. **Results:** The study population was primarily elderly (73 years, range 27-99 years) and male (79.7%). Of the 281 patients with ASB, 127 (45.2%) and 154 (54.8%) were untreated and treated, respectively. The incidence of UTI was 3% versus 1% (p = 0.41) at 30 days, 10% versus 12% (p = 0.61) at 6 months and 11% versus 12% (p = 0.94) at 12 months in the untreated and treated cohorts, respectively. There was no difference in admissions for UTI, sepsis from UTI or ADRs at 30 days. **Conclusion:** This study found no difference in the development of symptomatic UTI in veterans with untreated ASB compared to those treated with antibiotics. These findings align with current ASB guideline recommendations and support avoidance of unnecessary antibiotic use in the veteran population.

